# Relationship between myopia and diagnosis rates of dry eye disease and related indicators: a systematic review and meta-analysis

**DOI:** 10.3389/fmed.2025.1541304

**Published:** 2025-03-27

**Authors:** Kai Wu, Yunfeng Yu, Jian Shi, Huimei Chen, Canming Xie, Yu Tang, Xiaolei Yao

**Affiliations:** ^1^Department of Ophthalmology, The First Hospital of Hunan University of Chinese Medicine, Changsha, China; ^2^First Clinical College of Traditional Chinese Medicine, Hunan University of Chinese Medicine, Changsha, China; ^3^College of Integrated Traditional Chinese and Western Medicine, Hunan University of Chinese Medicine, Changsha, China

**Keywords:** myopia, emmetropia, dry eye disease, systematic review, meta-analysis

## Abstract

**Background:**

The association between myopia and dry eye disease (DED) has recently garnered considerable attention. This study aimed to compare the diagnosis rates of DED and its indicators between myopic and emmetropic patients to elucidate the association between myopia and DED.

**Methods:**

We retrieved relevant literature published through November 2024 from English databases, such as PubMed, Embase, the Cochrane Library, and Web of Science, as well as Chinese databases, such as the China National Knowledge Infrastructure, WanFang, VIP, and SinoMed. The studies were then screened for inclusion and exclusion criteria, and the basic information and outcome data of the included studies were recorded. The methodological quality of the included studies was assessed by the Joanna Briggs Institute. Finally, RevMan 5.3 was used to perform meta-, subgroup, and sensitivity analyses, as well as a publication bias assessment of the outcome data.

**Results:**

This study included 8 studies with a sample size of 14,232 patients. The meta-analysis showed that compared with emmetropic eyes, the diagnostic rate of DED in myopic eyes increased significantly, by 104% [odds ratio (OR) = 2.04, 95% confidence interval (CI) = 1.39–2.99, *P* = 0.0002, I^2^ = 91%], while the tear break-up time (BUT) was reduced significantly, by 6.31 s [weighted mean difference (WMD) = −6.31, 95% CI = −7.32 to −5.29, *P* < 0.00001, I^2^ = 0%]. However, there was no significant difference in the rate of positive corneal staining (OR = 2.53, 95% CI = 0.22–29.07, *P* = 0.46, I^2^ = 68%). Funnel plots showed a potential publication bias in DED diagnosis rate, rate of positive corneal staining, and BUT. An evaluation showed that the evidence quality of DED diagnosis rate, BUT and rate of positive corneal staining were extremely low.

**Conclusion:**

There were significant differences in the DED diagnosis rate and BUT between myopic and emmetropic patients, suggesting that myopia may be a potential risk factor for DED. The regular screening for DED should be a focus in myopic populations to improve detection and diagnosis rates.

**Systematic review registration:**

CRD42024611482, https://www.crd.york.ac.uk/PROSPERO/myprospero

## 1 Introduction

Dry eye disease (DED), a complex ophthalmic disease caused by insufficient tear secretion, excessive tear evaporation, and/or abnormal tear composition, is one of the most common ocular surface diseases encountered in clinical practice (1). Epidemiological surveys have shown that the incidence of DED ranges from 5 to 50%, which differs significantly by country and region (2). With the increasing popularity of electronic devices, the incidence of DED is increasing worldwide, positioning DED as a major threat to eye health (3). DED is primarily characterized by ocular pain and dryness, a foreign body sensation, and visual fatigue, frequently affecting patients' quality of life and work efficiency (4). Therefore, elucidating the main risk factors, etiology, and pathogenesis of DED is of great significance for its early diagnosis. Although the etiology and pathogenesis of DED have not yet been fully elucidated, sex, age, smoking, sleep quality, and excessive eye use are considered to be the main risk factors for DED (5–10).

The association between myopia and DED has recently garnered increasing attention. Myopia, one of the leading causes of visual impairment, is blurred vision caused by parallel light focusing in front of the retina after passing through the refractive system of the eye (11), and affects approximately half the world's population (12). Although there is no obvious direct association between myopia and DED, some reports have suggested that myopia may be a potential risk factor for DED (13–15). From an anatomical standpoint, the axial length of patients with myopia increases with the progression of the condition, which may lead to exophthalmos and expansion of the eye exposure area, in turn leading to thinning of the tear film and an increased risk of DED (16). A previous meta-analysis showed that the prevalence of subjective symptoms of DED in myopic individuals was 45.1%; however, this meta-analysis did not directly compare the differences in DED diagnostic rates between the myopic groups and healthy individuals (17). Additionally, given the lack of large sample sizes and multicenter clinical evidence, the specific impact of myopia on the risk of DED has not been well evaluated and summarized. Therefore, in this study, we performed a meta-analysis to compare the incidence of DED and DED-related indicators between myopic and emmetropic patients to elucidate the effects of myopia on DED.

## 2 Methods

### 2.1 Literature search

We searched English databases including PubMed, Embase, the Cochrane Library, Web of Science, and China National Knowledge, as well as Chinese databases including Infrastructure, WanFang, VIP, and SinoMed for relevant literature published through November 2024. The search field was set as “Title/Abstract” and the search formula was ([Myopia OR Myopias OR Nearsightedness OR Nearsightednesses] AND [Dry Eye Syndrome OR Dry Eye Syndromes OR Dry Eye Disease OR Dry Eye Diseases OR Dry Eye OR Dry Eyes]). No language restrictions were imposed.

### 2.2 Inclusion criteria and exclusion criteria

The inclusion criteria were as follows: (1) the study was of a cohort or cross-sectional study design; (2) participants included both emmetropic and myopic individuals; and (3) the primary endpoint was the diagnosis rate of DED, and the secondary endpoints were the rate of positive corneal staining and tear break-up time (BUT). Included studies reported relevant data for at least one endpoint.

The exclusion criteria were as follows: (1) duplicate published studies; (2) studies with missing baseline data; and (3) studies with unusable data.

### 2.3 Literature screening

All of the articles retrieved were imported into EndNote X9. First, we used the duplicate literature screening function of Endnote X9 to exclude duplicate studies through a review of the title, author, journal name, volume, issue number, and Digital Object Identifier (DOI) of each article. Next, the title and abstract of each article were reviewed, and articles that were not relevant to the research topic were excluded based on the inclusion criteria. Finally, the full texts of the remaining literature were reviewed to exclude irrelevant literature and finalize the included articles. Literature screening was independently completed and mutually proofread by KW and YY, and objections were adjudicated by XY.

### 2.4 Statistics of data

The basic characteristics and research data of the included studies were entered into a table created in Microsoft Excel 2010. These characteristics include the name of the first author, year of publication, country of publication, source of participants, study design, sample size, proportion of men, mean age, and age stage. The study data were defined as any measure related to the outcome. Baseline data and statistics were independently completed and mutually proofread by KW and YY, and any disagreements were adjudicated by XY.

### 2.5 Literature quality assessment

The Joanna Briggs Institute (JBI) was used to evaluate the quality of the included studies based on the following 10 modules: the basis for project selection, selection of study population, inclusion and exclusion criteria, characteristics of the sample, reliability and validity of the assessment tool, authenticity of the data, ethical issues, statistical methods, description of the research results, and elaboration of research value. Each module was scored from 0 to 2, and the higher the score, the higher the quality of the literature. The quality assessment of the literature was independently completed and mutually proofread by KW and YY, and any disagreements were adjudicated by XY.

### 2.6 Statistical analysis

RevMan software (version 5.3) was used to perform the meta-, subgroup, sensitivity analyses, as well as the assessment of publication bias. First, odds ratio (OR) and 95% confidence interval (CI) were used to evaluate the outcomes of dichotomous variables; weighted mean difference (WMD) and 95% CI were used to evaluate the results of continuous variables when the same measures were used; and otherwise, standardized mean difference (SMD) and 95% CI were evaluated. The *I*^2^ test was used to assess heterogeneity between the included studies. If the heterogeneity was small (*I*^2^ < 50%), a fixed-effects model was used; otherwise, a random-effects model was used. Statistical significance was set at *P* < 0.05.

Second, to explore and identify sources of heterogeneity at *I*^2^ ≥ 50%, we conducted subgroup analyses based on subject country, proportion of male individuals, and age stage. We subsequently conducted sensitivity analyses using the leave-one-out method to further explore the sources of heterogeneity and assess the robustness of the meta-analysis results. The meta-analysis results were considered robust when the pooled effect size was not significantly altered.

Funnel plots were used to assess the risk of bias for each outcome. Funnel plots were drawn with the OR or mean difference (MD) as the abscissa and SE(log[OR]) or SE(MD) as the ordinate to visually evaluate the distribution and symmetry of the results. If the scatter distribution on both sides of the funnel plot was asymmetrical, there was potential publication bias. Moreover, the Egger's test was used to quantify the severity of publication bias, with *P* < 0.05 indicating publication bias and a larger Egger intercept indicating more severe bias.

Finally, the quality of evidence for each outcome was evaluated according to the GRADE guidelines. The risks of bias, inconsistency, indirectness, imprecision, and publication bias were evaluated, and the quality of the evidence was classified as high, moderate, low, or very low.

## 3 Results

### 3.1 Literature screening

**Figure 1:**
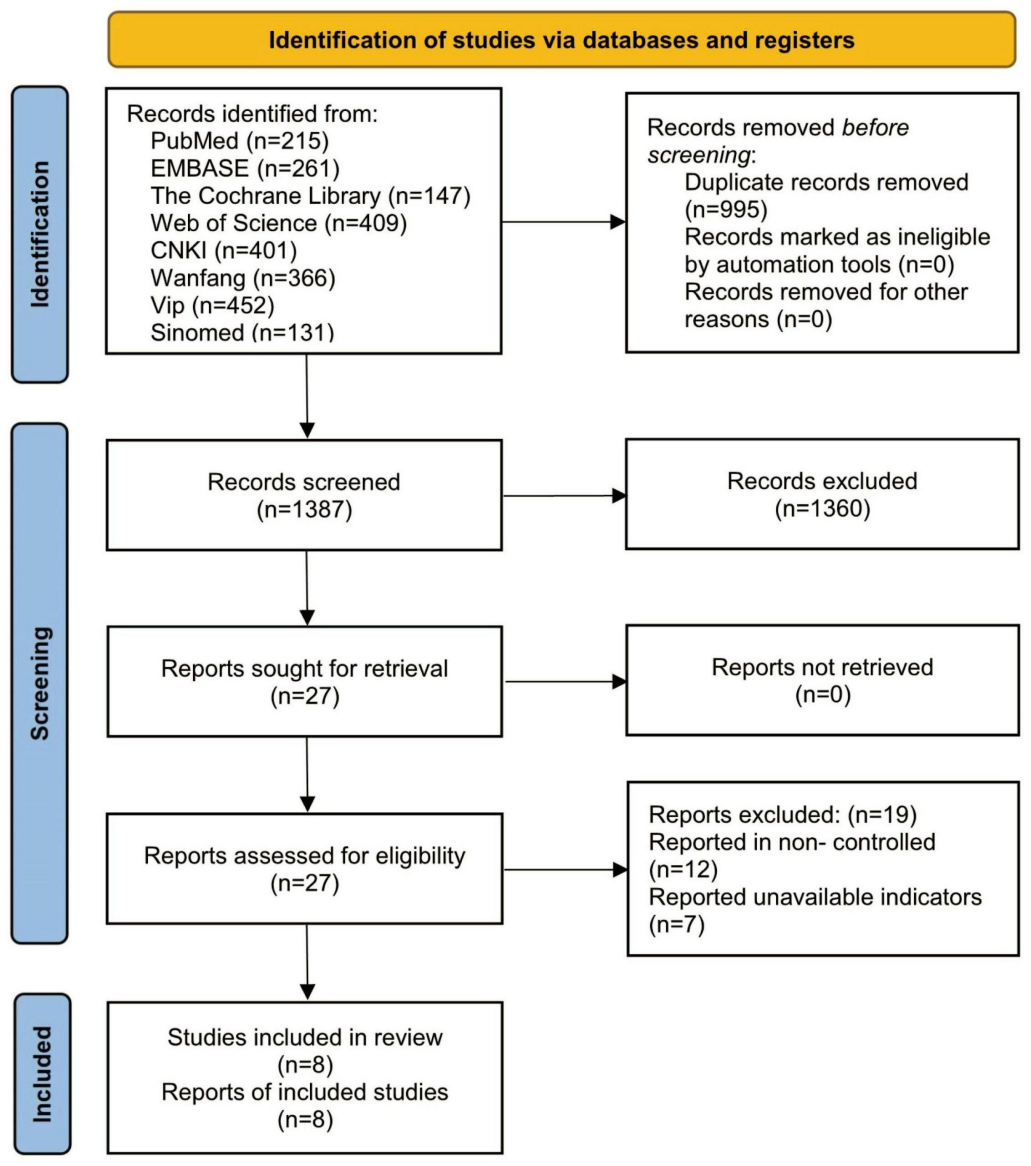
Literature screening flowchart.

A total of 2,382 articles were identified after the initial searches, of which 995 were excluded as duplicates, while 1,360 irrelevant articles were excluded during the review of titles and abstracts. We subsequently reviewed the full texts of 27 articles, of which 19 were excluded based on not meeting the inclusion criteria—12 non-controlled trials and 7 articles for which indicators were not available. After applying the inclusion/exclusion criteria, eight articles were included in this meta-analysis, as shown in Figure 1 (18–25).

### 3.2 Basic characteristics of the included studies

Of the eight included studies, six were conducted in China, one in Japan, and one in Turkey. All were retrospective and published between 2012 and 2024. A total of 14,232 eyes were included in these studies—11,619 myopic and 2,613 emmetropic eyes. The mean male-to-female ratio among the participants was 49.2%, and the mean age was 17.5 years (Table 1).

**Table 1:** Basic characteristics of included studies.

**Study**	**Country**	**Source of participants**	**Sample (E/C)**	**Male (%)**	**Age (years)**	**Age stage**	**JBI score**
Chang (18)	China	Chinese	133/31	52.9	11.1	Primary and secondary school students	14
Duan (19)	China	Chinese	1,068/212	50.3	17.8	High school students	15
Ibrahim (20)	Japan	Japanese	1,200/164	38.8	15.1	Primary and secondary school students	15
Ilhan (21)	Turkey	Turks	90/88	48.3	39.5	Adults	13
Lin (22)	China	Chinese	2,126/336	46.7	none	Primary and secondary school students	12
Lin (23)	China	Chinese	998/1,052	55.0	none	College students	9
Su (24)	China	Chinese	2,682/282	49.4	none	Middle and high school students	13
Zhang (25)	China	Chinese	3,322/448	50.8	none	High school students	14

E, experimental group; C, control group; JBI, Joanna Briggs Institute.

### 3.3 Literature quality assessment

The quality evaluation of the JBI literature showed that 2 articles (19, 20) scored 15 points, 2 (18, 25) scored 14 points, 2 (21, 24) scored 13 points, 1 (22) scored 12 points, and 1 (23) scored 9 points (Table 1).

### 3.4 Meta-analysis

#### 3.4.1 Diagnosis rate of DED

**Figure 2:**
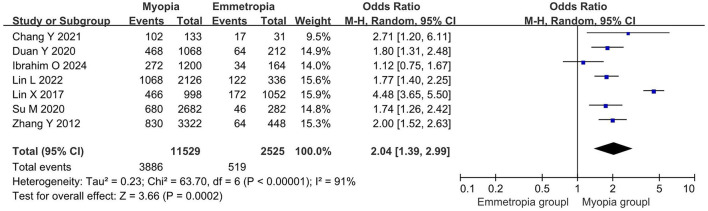
Meta-analysis of dry eye disease (DED) diagnosis rates in myopic and normal populations.

Seven studies reported DED diagnostic rates in myopic and emmetropic eyes, encompassing 11,529 myopic and 2,525 emmetropic eyes. The prevalence of DED in myopic eyes was 104% higher than that in emmetropic eyes (OR = 2.04, 95% CI = 1.39–2.99, *P* = 0.0002, *I*^2^ = 91%), as shown in Figure 2.

#### 3.4.2 Rate of positive corneal staining

**Figure 3:**
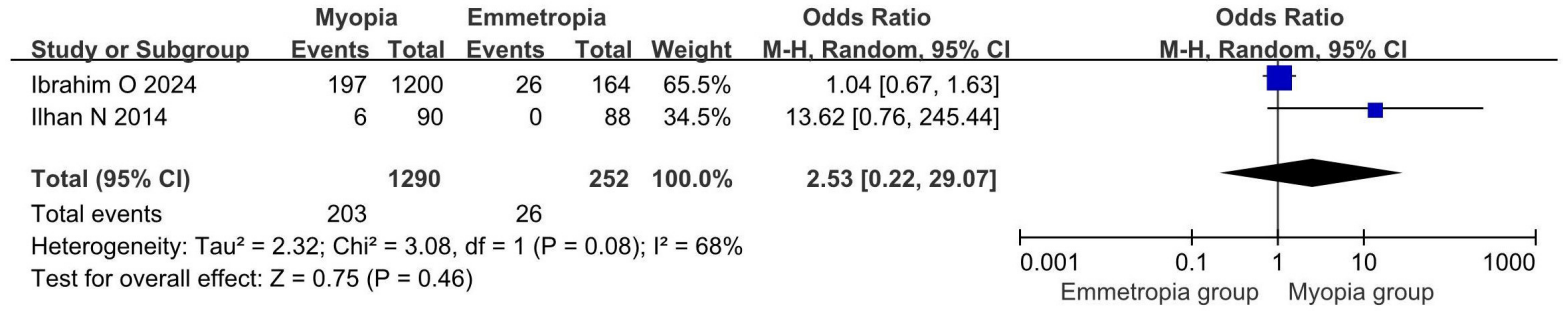
Meta-analysis of the rate of positive corneal staining in myopic and normal populations.

Two studies reported positive corneal staining rates in myopic and emmetropic eyes, encompassing 1,290 myopic and 252 emmetropic eyes. The meta-analysis showed that there was no significant difference in the rate of positive corneal staining between myopic and emmetropic eyes (OR = 2.53, 95% CI = 0.22–29.07, *P* = 0.46, *I*^2^ = 68%), as shown in Figure 3.

#### 3.4.3 BUT

**Figure 4:**
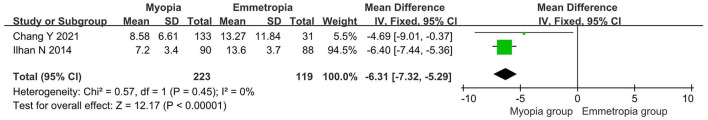
Meta-analysis of tear break-up time (BUT) in myopic and normal populations.

Two studies reported BUT in myopic and emmetropic eyes, encompassing 223 myopic and 119 emmetropic eyes. The meta-analysis showed that the BUT of myopic eyes was reduced significantly, by 6.31 s, compared with emmetropic eyes (WMD = −6.31, 95% CI = −7.32 to −5.29, *P* < 0.00001, *I*^2^ = 0%), as shown in Figure 4.

### 3.5 Subgroup analysis

Subgroup analyses based on the source of the participants, proportion of males, and age of the participants were used to explore the sources of heterogeneity in the DED diagnosis rates. However, in participant-based subgroup analyses, the Japanese subgroup contained only one eligible study. Similarly, in the male proportion-stratified subgroup analysis, the “male ratio ≥ 40%” subgroup also comprised a single study. Consequently, these subgroups were excluded from further comparative analysis due to insufficient statistical power stemming from the limited number of studies (*n* = 1) and small sample sizes, which precluded evidence-based interpretation.

**Figure 5:**
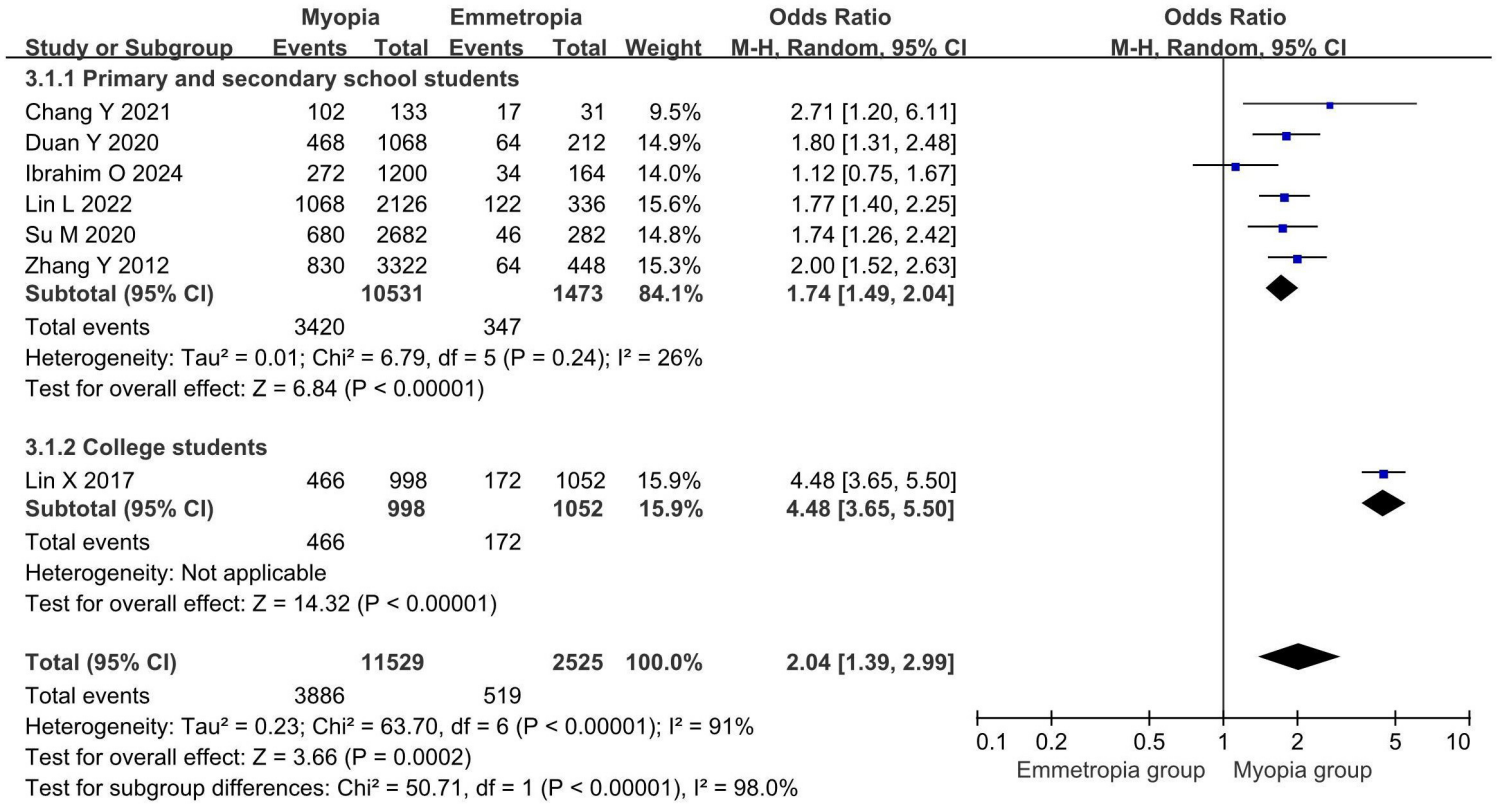
Subgroup analysis based on participant age stage.

Then, we divided the study participants into “primary and secondary school students” and “college students” subgroups, according to their age. The DED diagnosis rate of myopic eyes was significantly higher in the “primary and secondary school students” subgroup (OR = 1.74, 95% CI = 1.49–2.04, *P* < 0.00001, *I*^2^ = 26%), while the DED diagnosis rate of emmetropic eyes was significantly higher in the “college students” subgroup (OR = 4.48, 95% CI = 3.65–5.50, *P* < 0.00001, *I*^2^ = 0%). This subgroup analysis showed a significant decrease in heterogeneity, suggesting that the heterogeneity in DED diagnosis rates may be related to age (Figure 5).

### 3.6 Sensitivity analysis

The leave-one-out method was used to evaluate the sources of heterogeneity in the DED diagnosis rates and the robustness of the evaluation results. The results showed that the heterogeneity of the DED diagnosis rate was mainly derived from the study by Lin et al. After excluding that study, however, there was still a significant difference in DED diagnosis rate between the two groups (OR = 1.76, 95% CI = 1.54–2.01, *P* < 0.00001, *I*^2^ = 26%), indicating that the DED diagnosis rate results are robust. Sensitivity analyses were not performed because both the positive corneal staining rate and tear film BUT were included in the data from only two studies.

### 3.7 Bias of publication

**Figure 6:**
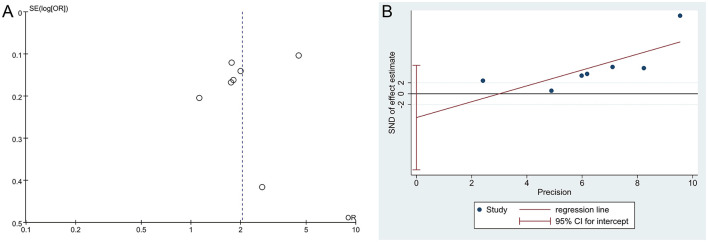
Funnel plots of publication bias. **(A)** The funnel plot of DED diagnosis rate, **(B)** Egger's test of DED diagnosis rate.

The funnel plot of the DED diagnosis rate showed an asymmetric distribution of scatter points on both sides, suggesting a potential publication bias; however, Egger's test indicated no publication bias in the result of DED diagnosis rate (*P* = 0.294), as shown in Figure 6. Additionally, the positive coronal staining rate and BUT were not suitable for publication bias assessment as they only included two studies.

### 3.8 Evaluation of evidence quality

The GRADE evidence quality evaluation showed that the evidence quality for DED diagnosis rate, BUT, and corneal staining was extremely low (Table 2).

**Table 2:** Evaluation of evidence quality.

**Outcome**	**Risk of bias**	**Inconsistency**	**Indirectness**	**Imprecision**	**Publication bias**	**Quality of evidence**
DED diagnosis	None	Serious	None	None	Serious	Low
Corneal staining	None	Serious	None	Serious	Serious	Very low
BUT	None	None	None	Serious	Serious	Low

DED, dry eye disease; BUT, break-up time.

## 4 Discussion

The association between myopia and DED is a topic of interest for ophthalmologists. Although a previous meta-analysis reported that the prevalence of subjective symptoms of DED in myopic individuals was as high as 45.1% (17), it did not directly compare the prevalence of DED between the myopic and emmetropic populations. To the best of our knowledge, this is the first meta-analysis to comprehensively evaluate the diagnosis rates of DED and DED-related indicators in myopic and emmetropic populations. The results of this meta-analysis showed that the DED diagnosis rate increased by 104% and BUT decreased by 6.31 s in the myopic group compared to the emmetropic group, whereas there was no significant difference in the rate of positive corneal staining.

When evaluating the primary endpoint, this meta-analysis showed that the DED diagnosis rates were 29.4 and 20.6% in the myopic and emmetropic groups, respectively, significantly higher in the myopic than in the emmetropic group. Our findings are supported by a systematic review by Zou et al. (17), which reported that the prevalence of subjective symptoms of DED in myopic patients was significantly higher than that in healthy individuals; however, they also reported a 45.1% prevalence of subjective symptoms of DED, which was much higher than the 29.4% reported in our study. This discrepancy may be related to the methods and criteria used to diagnose DED. In our meta-analysis, the diagnosis of DED in the included studies followed the 2013 Chinese expert consensus on the clinical diagnosis and treatment of dry eye and the criteria of the Asian Dry Eye Society (18–20). In contrast, Zou et al. (17) utilized a diagnostic approach for DED based on subjective symptoms, which partially increased the prevalence of DED. In a subsequent adjusted analysis, however, Zou et al. (17) excluded articles suspected of causing heterogeneity and found that the prevalence of DED symptoms was reduced to 34.7%, similar to the 29.4% reported in our study. Of note, our included studies addressed a variety of DED diagnostic criteria, including the 2013 Chinese Expert Consensus on Clinical Diagnosis and Treatment of Dry Eye, the 2017 Asian Dry Eye Association's criteria, and various rating scales. However, there are some differences between these diagnostic criteria and the classic TFOS DEWS II report. The TFOS DEWS II report (2017) established a key-standard framework emphasizing multi-dimensional assessment combining validated symptom scales (OSDI/5-Item Dry Eye Questionnaire) with objective metrics including non-invasive tear breakup time (NIBUT), osmolarity thresholds (≥308 mOsm/L in either eye or interocular difference >8 mOsm/L), and ocular surface staining [≥5 corneal spots, ≥9 conjunctival spots, or ≥2 mm length and ≥25% width lid margin involvement; (26)]. Compared to the TFOS DEWS II report, the 2013 Chinese Expert Consensus on the Clinical Diagnosis and Treatment of Dry Eye used by Chang et al. (18) and Duan et al. (19) includes the Schirmer I test as one of the key indicators, but lacks detailed descriptions of osmolarity and ocular surface staining. Additionally, the 2017 Asia Dry Eye Society's criteria used by Ibrahim et al. (20) requires a shorter BUT threshold (BUT ≤ 5 s), but still lacks detailed descriptions of osmolarity and ocular surface staining. Furthermore, five other studies depended exclusively on symptom-based questionnaires (OSDI, McMonnies, Salisbury, and Schaumberg scales) for diagnosis and severity grading, bypassing critical objective metrics like NIBUT and osmolarity (21–25). The inconsistency between these diagnostic methods and priority directly undermines the comparability of diagnosis and may lead to potential clinical heterogeneity. However, subgroup analysis to explore diagnostic criteria-related heterogeneity was precluded by insufficient stratification of diagnostic parameters across included studies. Such discrepancies underscore the need for standardized application of TFOS DEWS II protocols in future DED research to enhance cross-study validity.

Additionally, our subgroup analysis showed that the heterogeneity of the DED diagnosis rate was derived from age stage, and the DED diagnosis rate was found to be statistically significant in both the “primary and secondary school students” and “college students” subgroups, suggesting that the results are credible. Our sensitivity analysis also showed that the DED diagnosis rate results were robust. Interestingly, while the funnel plot suggested potential publication bias in DED diagnosis rates, Egger's regression test failed to confirm its presence (*P* = 0.294). This discrepancy may be attributed to the limited number of included studies (*n* = 7), as the reliability of funnel plot interpretation is inherently limited in small study populations. To address this methodological uncertainty, we performed a sensitivity analysis by excluding the under-powered study by Chang Y et al. The revised pooled estimates maintained a statistically significant elevation of DED diagnosis rates in the myopia group compared to the emmetropia group (OR = 1.98, 95% CI = 1.32–2.98, *P* = 0.001), demonstrating the robustness of the primary findings against potential publication bias. In conclusion, our meta-analysis found that myopia increased the diagnosis rate of DED by 104%, suggesting that myopia is a potential risk factor for DED.

When evaluating the secondary endpoints, this meta-analysis showed a significant (6.31 s) reduction in BUT in the myopic compared to the emmetropic group, with comparable rates of positive corneal staining. Similarly, a systematic review by Zou et al. (17) suggested that myopia reduces BUT in patients, supporting the findings of our meta-analysis. It is important to note that the systematic review by Zou et al. (17) included only studies by Ilhan et al. (21), whereas our meta-analysis included studies by both Ilhan et al. (21) and Chang et al. (18). These studies reported that the myopic group had shorter BUT than the normal group, suggesting that myopia is associated with reduced BUT and indirectly supporting myopia as a potential risk factor for DED. Additionally, both our meta-analysis and studies by Ibrahim et al. (20) and Ilhan et al. (21) showed that myopia was not associated with positive corneal staining, suggesting that the negative results of positive corneal staining are reliable. As only two studies included the rate of positive corneal staining, further subgroup and sensitivity analyses were not performed.

Among the included studies, Ilhan et al. (21) reported significant differences in the ocular surface disease index (OSDI) between the myopic and emmetropic groups. The OSDI is a questionnaire used to assess the impact of ocular surface diseases, such as DED, on quality of life, and is commonly used to assess the degree and impact of ocular surface disease symptoms in affected patients. Interestingly, a cross-sectional study by Ilhan et al. (21) showed a higher OSDI in the myopic than in the emmetropic group, and found that the Schirmer I test showed no statistical difference between the anesthetized and unanesthetized states. Although the significant difference in OSDIs between the myopic and emmetropic groups supports myopia as a potential risk factor for DED, the negative Schirmer I test results do not support this finding. Nevertheless, we cannot completely reject the difference between the myopic and normal populations in the Schirmer I test because Ilhan et al. (21) included only 178 samples; therefore, the negative result may have been the result of an insufficient sample size. Additionally, although the Schirmer I test can assess tear production, it cannot assess tear evaporation rate, implying that myopic individuals may be more susceptible to evaporative than aqueous DED. Unfortunately, as only Ilhan et al. (21) reported the results of both the OSDI and Schirmer I test, we were unable to perform a corresponding meta-analysis. We expect future studies will explore the differences between myopic and emmetropic patients using the OSDI and Schirmer I tests.

Myopia potentially increases the risk of DED by affecting the axial length, corneal surface curvature, and blink frequency. First, a change in axial length is a characteristic of patients with myopia, and axial length is positively correlated with the severity of DED (16). The increased axial length reduces the area of the eyelid covering the cornea, increasing the risk of exposing the ocular surface to a dry environment and leading to an increased risk of exposed-ocular surface diseases (27, 28). Second, the increase in axial length affects the corneal surface curvature, resulting in increased tension between the tear film and cornea, leading to uneven tear film distribution and DED symptoms (29). Third, most patients with myopia have a history of long-term electronic device use or close reading, which leads to a significantly lower blink frequency than that of the general population (30). Blinking plays an important role in maintaining ocular humidity, as while blinking, lipids and tears from the meniscus are redistributed onto the ocular surface, thereby reducing tear evaporation (31). Decreased blink frequency or quality can cause enhanced tear evaporation, tear film instability, and ocular surface inflammation, thereby increasing the risk of DED (32). In conclusion, myopia increases the risk of DED primarily through changes in the ocular anatomy and eye usage habits.

This study has some limitations. First, the included studies were cross-sectional and lacked causal inferences, which limited the quality of evidence in this meta-analysis. Second, with the exception of the study by Ilhan et al. (21), the research centers included in the literature were all located in Southeast Asia, and therefore lacks geographic diversity. The incidence of DED may vary greatly among myopic populations in different regions, considering the influence of environment, lifestyle, and medical care. Third, because most of the participants included in the literature were students, the study primarily explained the risks of myopia for DED in the student population; therefore, the results may not be applicable to other age groups. Fourth, because only two studies were included, the results of the positive corneal staining rate and BUT may have lacked precision. Fifth, the study could not interpret the association between the severity of myopia and DED due to the lack of data on myopia classifications. Therefore, we look forward to future studies exploring the causal effects of myopia on DED through multicenter cohort studies, and evaluating the influence of factors such as region, age, and degree of myopia on its causal effects on DED. Additionally, future studies should focus on the evaluation of the positive corneal staining rate, OSDI, BUT, and Schirmer I test to further enrich the clinical evidence. Moreover, it is recommended that future researchers conduct further searches of the gray literature and incorporate new public literature to more comprehensively evaluate the correlation between myopia and DED.

## 5 Conclusion

The present study showed significant differences in the DED diagnosis rate and BUT between myopic and emmetropic populations, suggesting that myopia may be a potential risk factor for DED. Therefore, it is recommended that the mild myopic group receive a tear secretion test, BUT and corneal fluorescent staining every two years, and the high myopic group receive a tear secretion test, BUT and corneal fluorescent staining every year.

## Data Availability

The original contributions presented in the study are included in the article/supplementary material, further inquiries can be directed to the corresponding author.
